# Biobased self healing waterborne polyurethane with vanillin derived dynamic Imine bonds for enhanced mechanical strength and performance

**DOI:** 10.1038/s41598-025-18911-0

**Published:** 2025-09-26

**Authors:** Ali Reza Banan, Seyed Mojtaba Keshavarz

**Affiliations:** Department of Organic Chemistry, Imam Hossein University, Tehran, Iran

**Keywords:** Waterborne polyurethane, Dynamic imine bond, Vanillin diol, Vanillin, Self-healing, Polytetrahydrofuran, Green chemistry, Polymer chemistry, Polymer characterization

## Abstract

Rising global interest in environmentally friendly, high-performance polymeric materials has accelerated the innovation of next-generation polyurethane systems. This research introduces a novel bio-based waterborne polyurethane, synthesized with a vanillin-derived green polyol chain extender featuring dynamic imine linkages. Vanillin diol (VAN-OH) was synthesized using a straightforward one-step condensation of vanillin and ethylenediamine, incorporating dynamic Schiff base functionality into the compound. The waterborne polyurethane (WPU) formulation was synthesized by combining the chain extender VAN-OH, dimethylolpropionic acid (DMPA), polytetrahydrofuran (PTHF), and isophorone diisocyanate (IPDI) in the proper molar ratios. The synthesis parameters were improved through a Design of Experiments (DoE) methodology to enhance mechanical characteristics. The WPU incorporating a vanillin-derived diol chain extender (WPU-VAN-OH) films exhibited a tensile strength of 12.8 MPa, three times greater than that of standard WPU at 4.3 MPa, and showed exceptional self-healing capabilities, completely mending surface scratches within 30 min at 80 °C by dynamic imine bond exchange. The material exhibited higher thermal stability, less water absorption (22.8% compared to 32.2% for WPU after 7 days), and superior adhesion to stainless steel (18.17 kgf/cm² versus 8.23 kgf/cm² for WPU). WPU-VAN-OH films presents a sustainable and efficient methodology for formulating polyurethanes characterized by high strength, self-healing properties, and environmental compatibility, appropriate for uses including protective coatings, advanced adhesives, and flexible elastomers.

**Supplementary Information:**

The online version contains supplementary material available at 10.1038/s41598-025-18911-0.

## Introduction

Waterborne polyurethanes (WPU), a class of versatile synthetic polymers, have taken center stage due to increasing awareness of environmental issues and are widely used in various applications across modern society ^1–3^. WPU dispersions consist of fine polyurethane particles suspended in a continuous water phase, forming a dual colloidal system^4,5^. The primary goal of producing WPU is to enhance its solubility and dispersibility in water by incorporating more hydrophilic groups. Since polyurethane is inherently water-insoluble, modifications to its chemical structure and polymer backbone are necessary. These adjustments not only improve water solubility but also enhance key mechanical and chemical properties, including tensile strength, surface characteristics, elongation, and resistance to solvents and chemicals^6,7^. WPU is widely used in various industries due to its biocompatibility^8^environmental-friendly^9^and outstanding properties^10^. This material has gained a special place in fields such as coatings^11^adhesives^12^elastomers^13^antimicrobial behaviors^14^and many other industrial applications due to its unique properties^15^. From protective coatings for surfaces to durable adhesives and flexible elastomers, WPU is recognized as a sustainable and efficient solution^16,17^.

The widespread use of polyurethane, with its rigid and permanent network structure, has contributed to resource waste and environmental pollution^18^. Covalent adaptable networks (CANs) challenge the traditional belief that coatings are non-recyclable or non-reprocessable^19^. By utilizing dynamic covalent bond exchange, these coatings allow for reusability and degradability, making them more sustainable and versatile^20^. A wide range of dynamic exchange reactions have been employed to develop CANs, including transesterification^21^transamination of vinyl-urethane^22^disulfide exchange^23^olefin metathesis, Diels-Alder addition reactions^24^dioxa-borolane metathesis^25^imine reactions^26^and more^27^. The mechanism of action of CANs can be divided into two main categories: associative cross-link exchange mechanisms and dissociative mechanisms^28^. Among these, imine exchange is considered a special covalent reversible interaction due to its unique dynamic mechanisms^29^. These mechanisms include the reaction of an amine group with an aldehyde group, reversible imine bond exchange, and the hydrolysis of a Schiff base^26^. These features provide polymers based on Schiff bases with multifunctionality, making them capable of recyclability, reprocessing, re-welding, self-healing, and controlled degradability^30^. These advancements not only provide a solution to reduce the environmental impacts of traditional polyurethanes but also lay the foundation for the development of smart and sustainable materials for future industrial applications^31^. Zang and colleagues^32^ introduced urea-based polyurethane networks derived from castor oil using 4-aminophenyl disulfide and castor oil-based polyurethane precursors. These networks can be easily reprocessed, thanks to the dynamic aromatic disulfide bond exchange, and they exhibit superior mechanical properties. However, the maximum tensile strength of these materials is less than 12.8 MPa, and the cost of 4-aminophenyl disulfide is very high, making commercialization difficult. Therefore, the production of castor oil-based polyurethanes that are low-cost, provide excellent mechanical properties, and are reprocessable remains a significant challenge.

Vanillin, a high-yield biomonomer derived from lignin, offers significant potential for the production of cost-effective and high-performance biopolymers due to its unique structure and active functional groups^33–35^. Its distinctive chemical structure, which includes hydroxyl and aldehyde groups at the para positions on the benzene ring^36,37^enables extensive design and modification possibilities. In simpler terms, vanillin provides an inexpensive and practical alternative to petroleum-based raw materials for producing target products, which has attracted considerable attention from researchers. Although new bio-based polyurethanes have been synthesized using vanillin and lignin, these materials face challenges, including complex production processes and poor mechanical properties^38–40^.

This study introduces the development of a novel vanillin-based diol featuring dynamic Schiff base linkages at its terminal ends, enabling reversible covalent interactions within the polymer network. Leveraging vanillin, a low-cost, bio-derived compound, as a renewable building block, we synthesized a WPU system that integrates this functional diol alongside polytetrahydrofuran (PTHF) and isophorone diisocyanate (IPDI). The inclusion of Schiff base chemistry imparts self-healing ability and reprocessability to the resulting material while also enhancing its mechanical integrity. To optimize the formulation, we employed a systematic Design of Experiments (DoE) approach. The optimized WPU formulation not only exhibited superior tensile strength and thermal stability but also demonstrated excellent self-healing behavior under mild thermal conditions. The following sections discuss the detailed synthesis process, characterization techniques, and performance evaluations.

## Experimental

### Materials

Polytetrahydrofuran (PTHF, Mn = 1000 g/mol) was sourced from Sigma-Aldrich. Isophorone diisocyanate (IPDI, 99%) was obtained from Rongrong Chemical Ltd. Vanillin (VAN, 98%) and ethylenediamine (EDA, 99%) were sourced from Sigma-Aldrich. Dimethylolpropionic acid (DMPA, 98%), triethylamine (TEA, 98%), and dibutyltin dilaurate (DBTDL, 98%) were all purchased from Merck. Ethylene glycol and ethanol (analytical grade, purity ≥ 99.8%) were obtained from Merck, and ultrapure water was used for all procedures.

### Synthesis of Vanillin diol containing dynamic Imine bond (VAN-OH)

In the first step, 5 g (32 mmol) of vanillin (VAN) was dissolved in 5 mL of ethanol and added to a 100 mL three-necked flask equipped with a reflux condenser. Ethylenediamine (1 g, 16 mmol) was carefully added dropwise to the reaction mixture, resulting in the immediate formation of a yellow precipitate. The mixture was then heated to 55 °C and refluxed under magnetic stirring for duration of 8 h to ensure complete reaction. Following the washing process with ethanol and water, the yellow precipitate was obtained using filtration. Finally, the product was dried under vacuum at 70 °C for 24 h to a constant weight, yielding a yellow powdery substance, which was named Van-OH (5.5 g, yield: 92.62%, melting point: 232–233 °C)^41,42^.

### Synthesis of dynamic Imine bond waterborne polyurethane

**Fig. 1:**
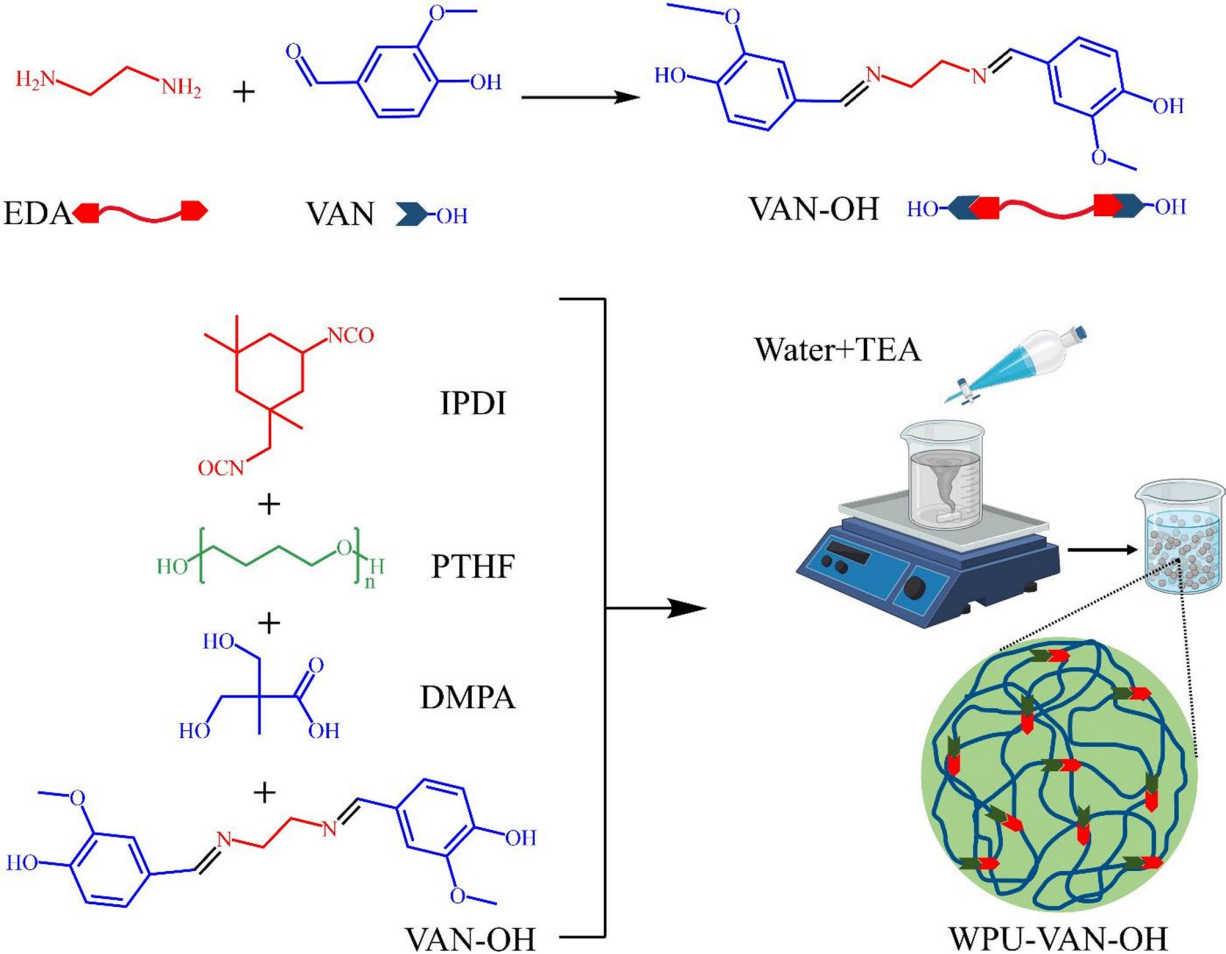
Synthesis route for waterborne polyurethane containing vanillin-derived dynamic imine bonds.

A solvent-free synthesis method for WPU was devised, wherein PTHF 15 g (15.9 mmol) and IPDI 6 g (27 mmol) were introduced into a three-necked 100 mL reactor. Subsequently, DBTDL (20 µL) was added, and the mixture was heated to 70 °C. The blend was mechanically stirred under a nitrogen atmosphere for 4 h. Then, DMPA 1 g (7.5 mmol) and VAN-OH 1.2 g (3.6 mmol) were incorporated into the mixture, which was stirred at 80 °C for another 4 h until the NCO concentration reached its theoretical value. The resulting NCO-terminated polyurethane prepolymer was cooled to 40℃. A mixture of distilled water and triethanolamine (TEA) was added gradually via a dropping funnel to the reaction system, which was maintained at 40 °C and continuously stirred mechanically for one hour (Fig. 1). This process ensured the effective neutralization of the carboxylic acid groups present in DMPA. Ultimately, a milky dispersion of WPU-VAN-OH was successfully obtained.

### Optimization of reaction parameters by DoE approach

The influence of various factors and the key parameters impacting the quality of WPU was evaluated using the DoE approach. The Taguchi methodology was employed, utilizing factorial design to systematically arrange experiments through an orthogonal array framework. We found that four primary factors influenced the stress levels in WPU: the amounts of DMPA, VAN-OH, IPDI, and PTHF. The specific values for each factor were thoughtfully selected based on insights from prior research^43,44^. To ensure a clear and accurate representation of value distribution, three distinct levels were assigned to each factor, as detailed in Table 1.

**Table 1:** Levels of each factor and their corresponding values for the screening process parameters.

Factor (g)	Symbol	Level 1	Level 2	Level 3
Dimethylolpropionic acid	DMPA	0.5	1	1.5
Vanillin diol	VAN-OH	0.5	1.2	2.4
Isophorone diisocyanate	IPDI	3	6	9
Polytetrahydrofuran	PTHF	7	15	30

A total of 9 experiments were carried out using a conventional orthogonal array designed according to the Taguchi method. The collected data was statistically analyzed using Minitab, ensuring reliable and accurate results. Three levels were utilized to evaluate the effect of each condition with respect to the selected variables. The systematic execution of these tests and a comprehensive analysis of the results determined that formulation number 10 was the optimal choice. This formulation specifies the most effective levels for each factor involved in the production of WPU, ensuring optimal performance and quality, and also investigates the effect of VAN-OH as a chain extender on the polyurethane chain. An additional experiment, designated as Experiment 11, was carried out. In this experiment, the optimal values for all factors were utilized for the synthesis of waterborne polyurethane (WPU), with the exception of the chain extender VAN-OH, which was excluded by setting its value to zero. The waterborne polyurethane was then prepared based on these conditions to evaluate the impact of excluding the chain extender on the final properties of the polymer, as presented in Table 2.

### Instrumentations and measurements

The particle size and distribution of the emulsion were analyzed using dynamic light scattering (DLS) with a Brookhaven 90 Plus Laser Particle Analyzer. Samples were diluted in deionized water at room temperature, and each measurement was repeated three times to ensure accuracy. Fourier-transform infrared spectroscopy (FT-IR) spectra were recorded in ATR mode within a wavenumber range of 650–4000 cm⁻¹, at a resolution of 8 cm⁻¹, using thin polymer films for analysis. The contact angle of the film was measured using a Contact Angle Goniometer (DSA30, Kruss Co.), and surface energy was determined through contact angle experiments with water and ethylene glycol, following the Owen-Wendt theory. Three independent measurements were conducted for each sample to confirm the reliability of the results, enabling a detailed evaluation of liquid-solid interactions.

Tensile testing of the films was performed using a universal testing machine (Instron 3365 tensile tester) at a crosshead speed of 50 mm/min and a temperature of 25 °C, adhering to ASTM D638 standards. Each sample was tested in triplicate, and the optimal value from these tests was selected for analysis. Uniaxial tensile tests were carried out on free-standing films at 25 °C. Engineering stress–strain data were continuously recorded (time, extension, strain, force, stress). The tensile strength (σ_b_) was defined as the maximum engineering stress, while the elongation-at-break (ε_b_) was taken as the strain at the fracture point. The fracture point was identified as the final drop of the force signal below 5% of its maximum after smoothing to reduce noise. Reported values represent the average of replicate measurements. Thermogravimetric analysis (TGA/DTG) was carried out using a Pyris thermogravimetric analyzer. Film samples weighing 7–8 mg were placed in platinum pans and heated under a nitrogen atmosphere at a rate of 10 °C/min from 25 °C to 600 °C. Scanning electron microscopy (SEM) was employed to analyze the surface morphology of WPU-VAN-OH films, which revealed structural changes during the synthesis process.

The water absorption of WPU and WPU-VAN-OH films was evaluated by immersing dried film samples in water for one week. Samples were removed daily, blotted dry, and weighed. Water absorption (W) was calculated using Eq. 1:

1 $$\:{\text{W}}\left( \% \right) = \frac{{{\text{W}}_{{\text{t}}} - {\text{W}}_{0} }}{{{\text{W}}_{0} }} \times 100$$

Where *W*_0_​is the initial mass of the film and *W*_*t*​_ is the mass at time *t*.

The total solids content (TSC) of the WPU and WPU-VAN-OH mixture was determined according to ISO 124:1997. One gram of a waterborne polyurethane solution was measured before being dried in an oven at 70 °C, and its weight was recorded again after the solvent had evaporated. The procedure was repeated until a constant weight was achieved, and TSC was calculated based on the weight difference before and after drying. Dispersion stability was assessed by visually examining the physical appearance of samples stored undisturbed in sealed clear containers for four months.

Adhesion properties of WPU and WPU-VAN-OH films applied to stainless steel substrates were evaluated using cross-cut adhesion tests (ASTM D3359) and pull-off strength (ASTM D4541). For the cross-cut test, a multi-blade cutting tool (Model 0302001, Neurtek Instruments S.A.) was employed to make six parallel incisions, followed by perpendicular cuts, creating a grid pattern on the WPU and WPU-VAN-OH films. Tesa adhesive tape was applied to the grid and pulled off, allowing the number of removed 1 × 1 mm squares to be counted. Three replicates were performed, and the results were averaged, with adhesion rated according to the ASTM D3359 scale.

For the pull-off strength analysis, the Positest-AT-Automatic (Defelsko Co., NY) was employed to evaluate the WPU and WPU-VAN-OH films. A mold with an area of 12.7 mm² was used, and surface contaminants were removed through a triadic cleaning process involving a detergent solution. A stud was bonded to the surface of the polyurethane film using a thermally curable epoxy adhesive, and the pull-off strength values were subsequently measured. This technique offered a thorough evaluation of the adhesion characteristics of the coatings on stainless steel substrates.

The self-healing properties of WPU and WPU-VAN-OH films were assessed using visual inspection of coated mild steel plates. Films with an approximate thickness of 70 μm were deliberately incised using a scalpel blade to induce controlled damage. The scratched areas were meticulously analyzed pre- and post-thermal treatment utilizing a Zeiss Primostar optical microscope, fitted with an AmScope 423x eyepiece camera, to get high-resolution photographs of the affected regions. During optical microscopy observations, the samples were fixed on the stage to maintain identical coordinates. For both WPU and WPU-VAN-OH, red circle markers were added to the images to clearly indicate the same scratch location at different time intervals. This method facilitated accurate observation of the healing process and comparison of surface restoration between the two coats.

## Results and discussion

### Design of experiments for synthesized films

Experimental design is a key area of focus in various industries and laboratory settings today. Utilizing DOE methods enables the identification of the interactive effects of different factors that influence measurement outcomes. In this research, the Taguchi method was employed to evaluate the factors affecting the stress properties of WPU-VAN-OH films. The data collected through this approach were analyzed to determine the most influential factors and to define the optimal levels for each factor in relation to the objective function, as summarized in Table 2.

**Table 2:** Formulation screening using Taguchi design.

Test number	Factors				Results
	DMPA	VAN-OH	IPDI	PTHF	Stress [MPa]
1	0.5	0.5	3	7	5.1
2	0.5	1.2	6	15	11.8
3	0.5	2.4	9	30	5.2
4	1.0	0.5	6	30	9.3
5	1.0	1.2	9	7	9.9
6	1.0	2.4	3	15	10.8
7	1.5	0.5	9	15	6.1
8	1.5	1.2	3	30	11.5
9	1.5	2.4	6	7	6.3
10*	1.0	1.2	6	15	12.8
11*	1.0	0.0	6	15	4.3

*This indicates an optimized formulation; all component values are measured in grams, and stress values represent tensile strength in MPa.

**Fig. 2:**
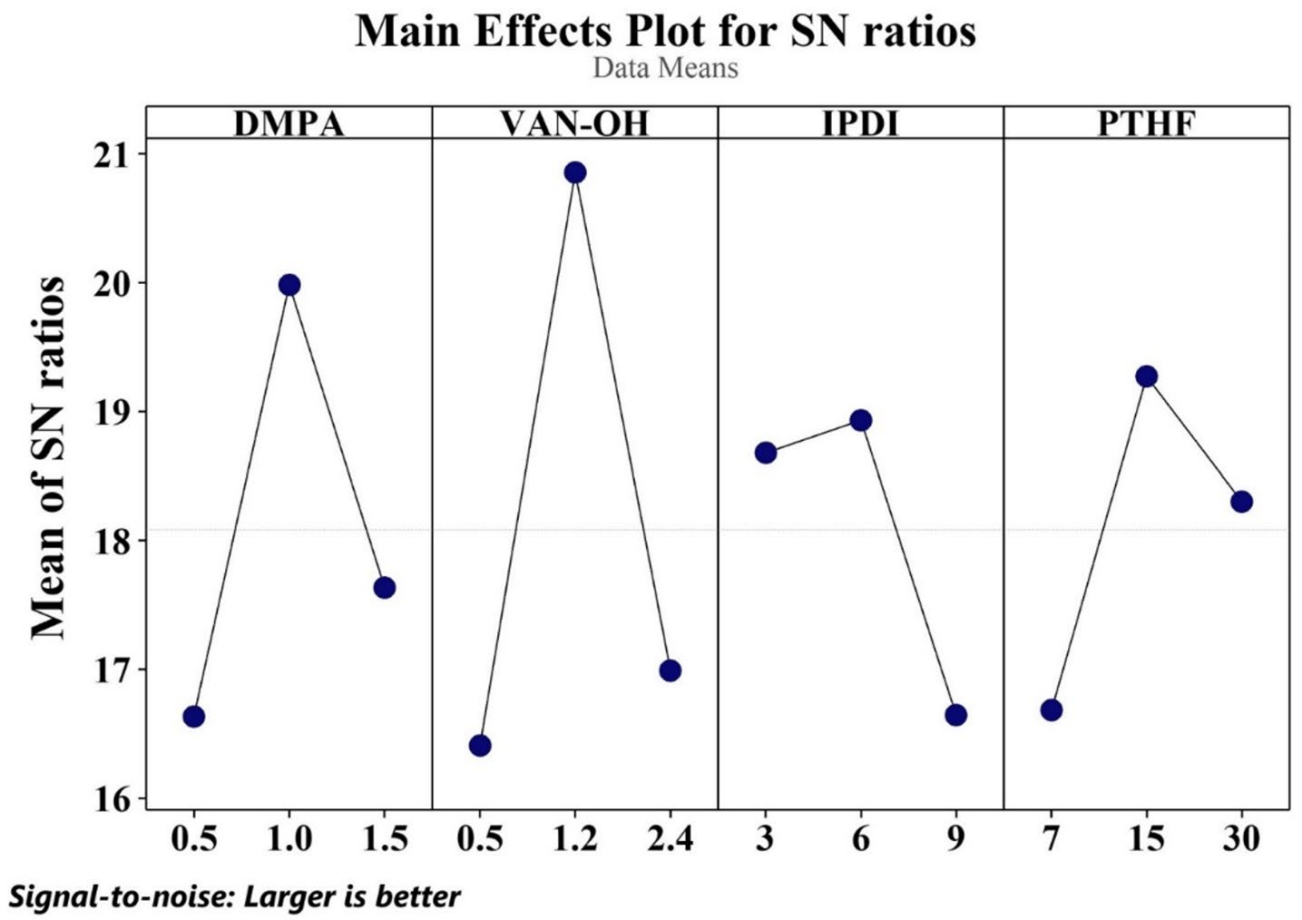
Optimal values of factors determined by signal-to-noise ratio curves.

Taguchi analysis revealed that the most influential factor affecting the trial outcome was the quantity of VAN-OH, with 1.2 g (level 2) identified as the optimal value. The second most significant factor was the amount of DMPA, with level 2 (1 g) deemed optimal. PTHF, at 15 g (level 2), and IPDI, at 6 g (level 2), were ranked third and fourth, respectively, for their impact on the tensile strength of the polymer film. To confirm the reliability of these findings, new samples, labeled Sample 10 and Sample 11, were prepared using the optimal levels of all identified parameters. Sample 11 serves as the primary reference, with VAN-OH set to zero, while Sample 10 illustrates the effect of VAN-OH on modifying the polymer’s properties, as shown in Fig. 2.

### Response surface methodology

**Fig. 3:**
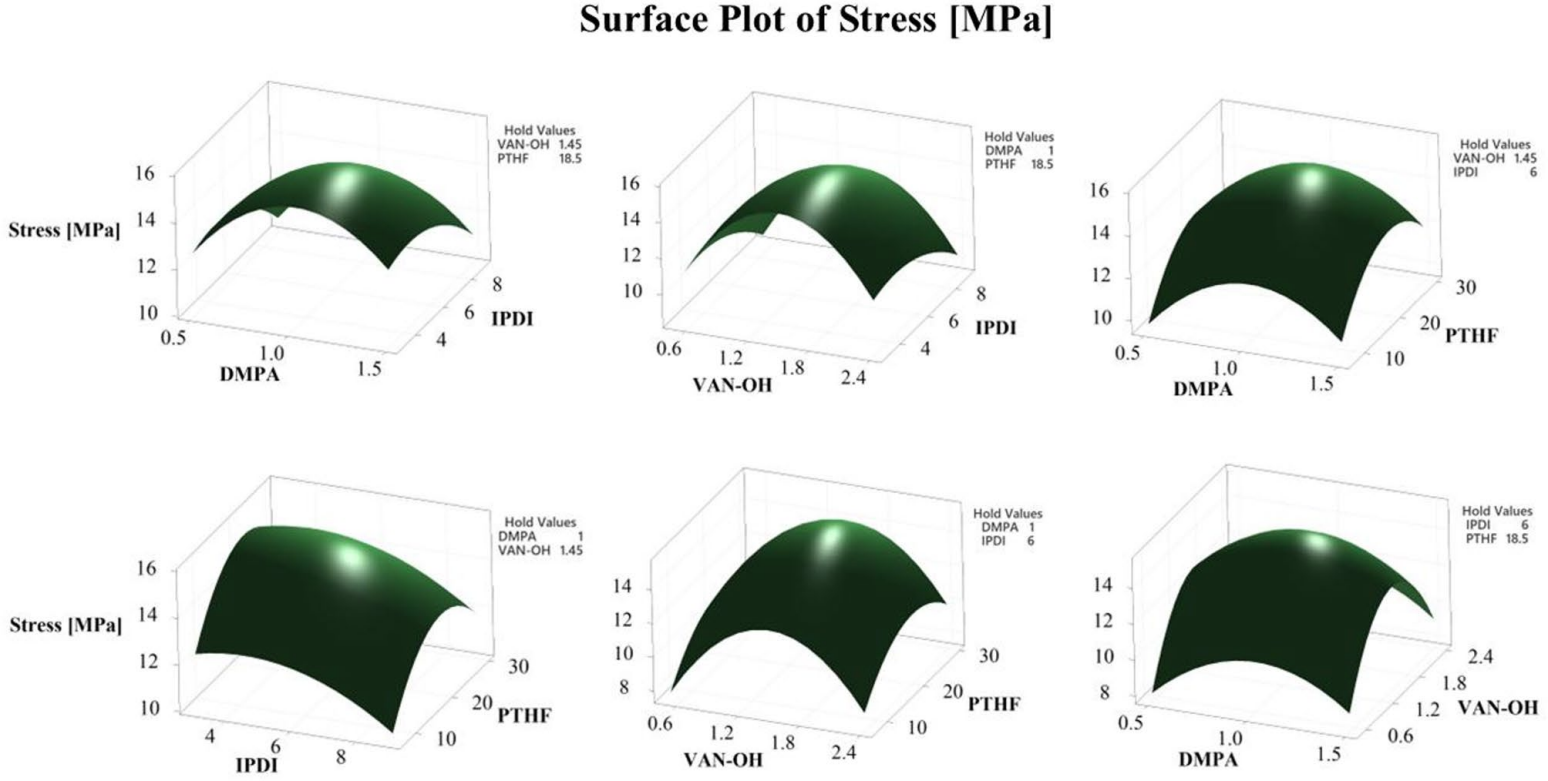
Three-dimensional surface plot of the response surface model illustrating the effect of two factors on the tensile strength of the WPU-VAN-OH film.

Response Surface Methodology (RSM) was applied to analyze the relationship between critical synthesis parameters and the mechanical performance of WPU films^45^. In this statistical framework, IPDI, PTHF, VAN-OH, and DMPA were defined as independent variables, while the film’s tensile strength served as the response (dependent variable). Three-dimensional (3D) plots generated through RSM visually clarified the interplay between these variables and their collective impact on film stress. Each plot isolated two variables, holding the remaining two constant, to dissect both individual and synergistic effects on mechanical properties. As shown in Fig. 3, the tensile strength peaks sharply when variables align with their optimal levels, demonstrating the method’s effectiveness in identifying ideal conditions. This visualization underscores how subtle adjustments in IPDI, PTHF, VAN-OH, and DMPA concentrations critically govern the final performance of WPU-VAN-OH films.

**Fig. 4:**
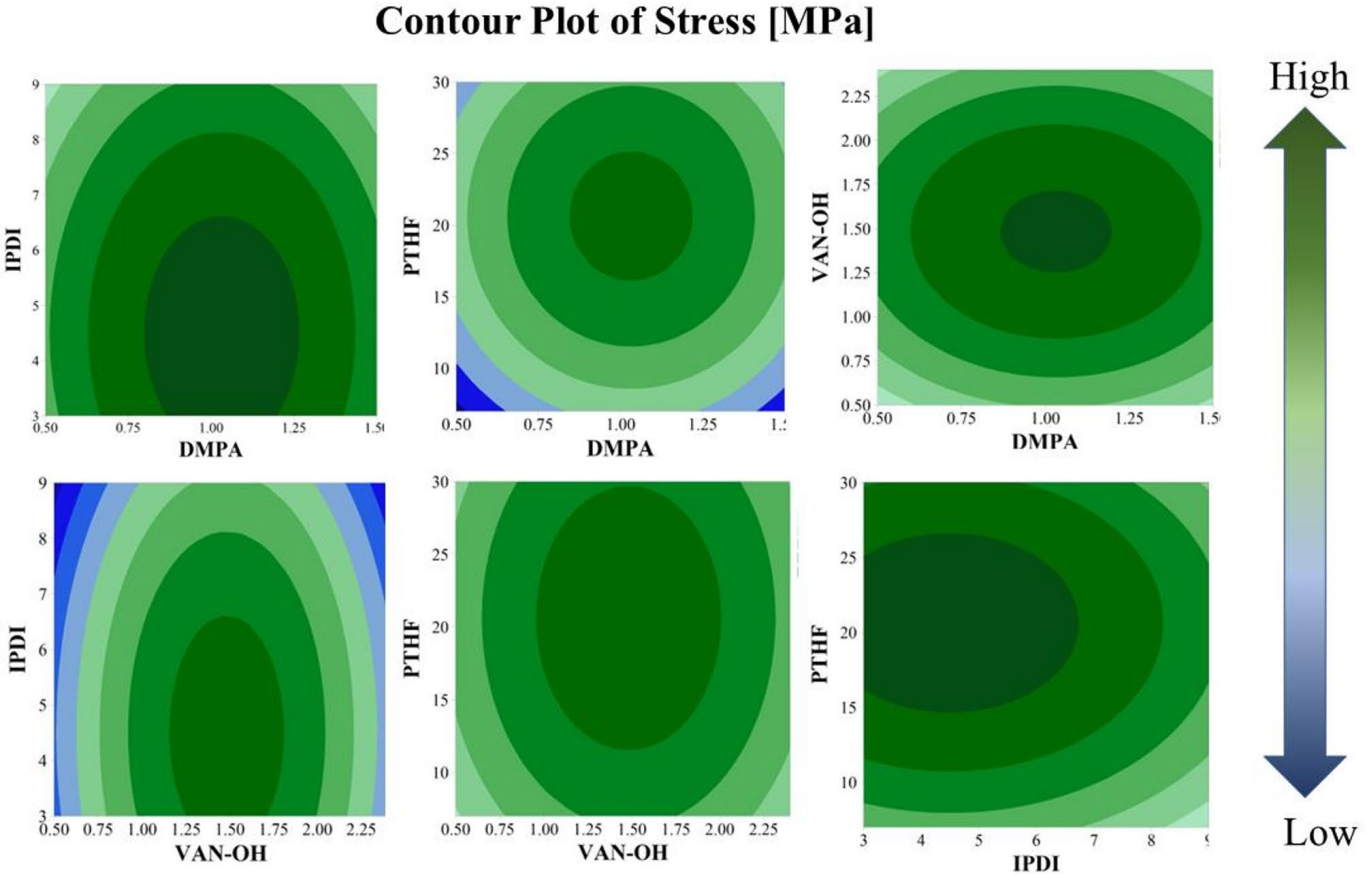
Contour plot of the response surface model showing the effect of two factors on the tensile strength of the WPU-VAN-OH film.

Figure 4 displays two-dimensional (2D)contour plots derived from RSM predictive models, visualizing the interplay of synthesis parameters and their effects on the mechanical resistance of WPU-VAN-OH films. The color gradient (ranging from minimal to maximal values) elucidates how varying combinations of parameters influence film performance. Notably, increasing VAN-OH to level 2 significantly enhances film tensile strength^46^. This improvement arises from VAN-OH’s role in extending polymer chain length. However, further increases to level 3 result in a decline in mechanical resistance, likely due to phase separation within the WPU-VAN-OH film structure.

Complementarily, optimizing DMPA concentration (level 2) strikes equilibrium between chain flexibility and structural integrity, thereby enhancing overall mechanical properties. The contour plots also reveal synergistic interactions between VAN-OH and DMPA: While VAN-OH, as a chain extender, alters the physical properties of the film; DMPA contributes acidic groups that stabilize the polymer matrix, collectively improving stress resistance and durability. These visual insights provide a strategic framework for fine-tuning parameters to achieve target mechanical properties in WPU-VAN-OH films.

### Properties of WPU and WPU-VAN-OH

WPU and WPU-VAN-OH were efficiently synthesized via step-growth polymerization. Table 2 presents the different formulations along with the effects of various parameters on the mechanical properties, which ultimately led to the development of the optimal product. Visually, WPU appeared milky with total solids content (TSC) of 38%, while WPU-VAN-OH had a light-yellow color and a lower TSC of 33% (Supporting information, Figure S1). This difference indicates variations in phase separation and dispersion stability caused by the addition of VAN-OH^47^. Although both WPU and WPU-VAN-OH were synthesized under comparable stoichiometric conditions, the VAN-OH-modified system exhibited a TSC about 5% lower. The incorporation of aromatic/imine functionalities can reinforce inter-chain associations and modulate dispersion/film-formation behavior in water, which is known to affect water uptake and the apparent TSC after identical drying protocols. Similar sensitivities of solids content to polyurethane structure, ionic content, and chain extension strategy have been reported^48–51^. The particle size of WPU is influenced by several critical factors, including the NCO/OH ratio, the amount and type of chain extender, the molecular weight and nature of the polyol, and the degree of neutralization^52^. The particle sizes of WPU and WPU-VAN-OH were compared, revealing an increase from 73 nm to 81 nm upon the incorporation of VAN-OH, a hydroxyl-functionalized chain extender. This rise in particle size can be attributed to the role of the chain extender in increasing the molecular weight of the polymer chains, which leads to greater entanglement and reduced mobility during dispersion formation.

The addition of VAN-OH also alters the hydrophilic-lipophilic balance of the polymer. If the chain extender incorporates polar groups, it could increase hydrophilicity, which might destabilize the emulsion and lead to partial aggregation^53,54^. Conversely, if VAN-OH incorporates hydrophobic segments, it may promote phase separation and coalescence of particles. Furthermore, the increased molecular weight due to chain extension raises the viscosity of the prepolymer phase, resisting shear forces during emulsification and resulting in larger droplets.

The stability of aqueous polyurethane dispersions is a critical determinant of their industrial viability and processing reliability^55^. Systems with particle sizes below 100 nm are highly valued for their storage stability and elevated surface energy, which ensure uniform film formation and consistent performance. As shown in Table 3, both WPU and WPU-VAN-OH dispersions exhibited excellent stability over three months, remaining visually homogeneous and free from sedimentation throughout the period. Over the initial three months, WPU maintained exceptional colloidal integrity, with particle size increasing only marginally from 73 nm to 85 nm, accompanied by a PDI consistently ≤ 0.15 (Supporting information, Figure S2). This narrow PDI range indicated a stable and uniform particle size distribution. The result from WPU-VAN-OH showed a particle size evolution, escalating from 81 nm (PDI = 0.18) in the first week to 91 nm (PDI = 0.22) by the third month (Supporting information, Figure S3). Despite this gradual aggregation, the dispersion retained functional stability for practical applications within the three-month window.

By the fourth month, both formulations displayed obvious signs of instability, including visible aggregation and sedimentation. Specifically, WPU-VAN-OH showed faster degradation, likely due to the hydrophobic groups in its VAN-OH chain extender. These groups upset the colloidal stability over time, inducing particle aggregation. In contrast, WPU showed less degradation, likely due to the absence of hydrophobic components, although other factors in its formulation may also have contributed to its instability^41^.

**Table 3:** The properties of the WPU and WPU-VAN-OH dispersions.

Sample	TSC	Stability			Average particle size (nm)					
	%	1 week	3 months	4 months	1 week		3 months		4 months	
					DLS	PDI	DLS	PDI	DLS	PDI
WPU	25	Stable	Stable	Unstable	73	0.1	85	0.14	–	–
WPU-VAN-OH	31	Stable	Stable	Unstable	81	0.18	91	0.22	–	–

### The characterization of polyurethane films

The FT-IR spectra of PTHF reveal distinct peaks at 1105 cm⁻¹, attributed to C-O-C stretching vibrations, confirming the presence of ether bonds. Bands at 1347 cm⁻¹ and 1440 cm⁻¹ correspond to C-H bending in aliphatic CH₂-groups, while bands at 2856 cm⁻¹ and 2938 cm⁻¹ are assigned to C-H stretching vibrations in methylene groups, collectively validating the ether linkages and methylene backbone in PTHF^56,57^. For DMPA, a broad band at 3353 cm⁻¹ signifies O-H stretching vibrations of hydroxyl groups, and a sharp band at 1681 cm⁻¹ corresponds to carbonyl (C = O) stretching in carboxylic acid functionalities. In the spectra of vanillin-functionalized hydroxyl compound (VAN-OH), a prominent band at 3097 cm⁻¹ confirms O-H stretching vibrations, while the band at 1633 cm⁻¹ is characteristic of imine (C = N) bond formation. Additionally, a band at 1028 cm⁻¹ indicates C-O stretching vibrations of the ether groups in VAN-OH^41,42^.

**Fig. 5:**
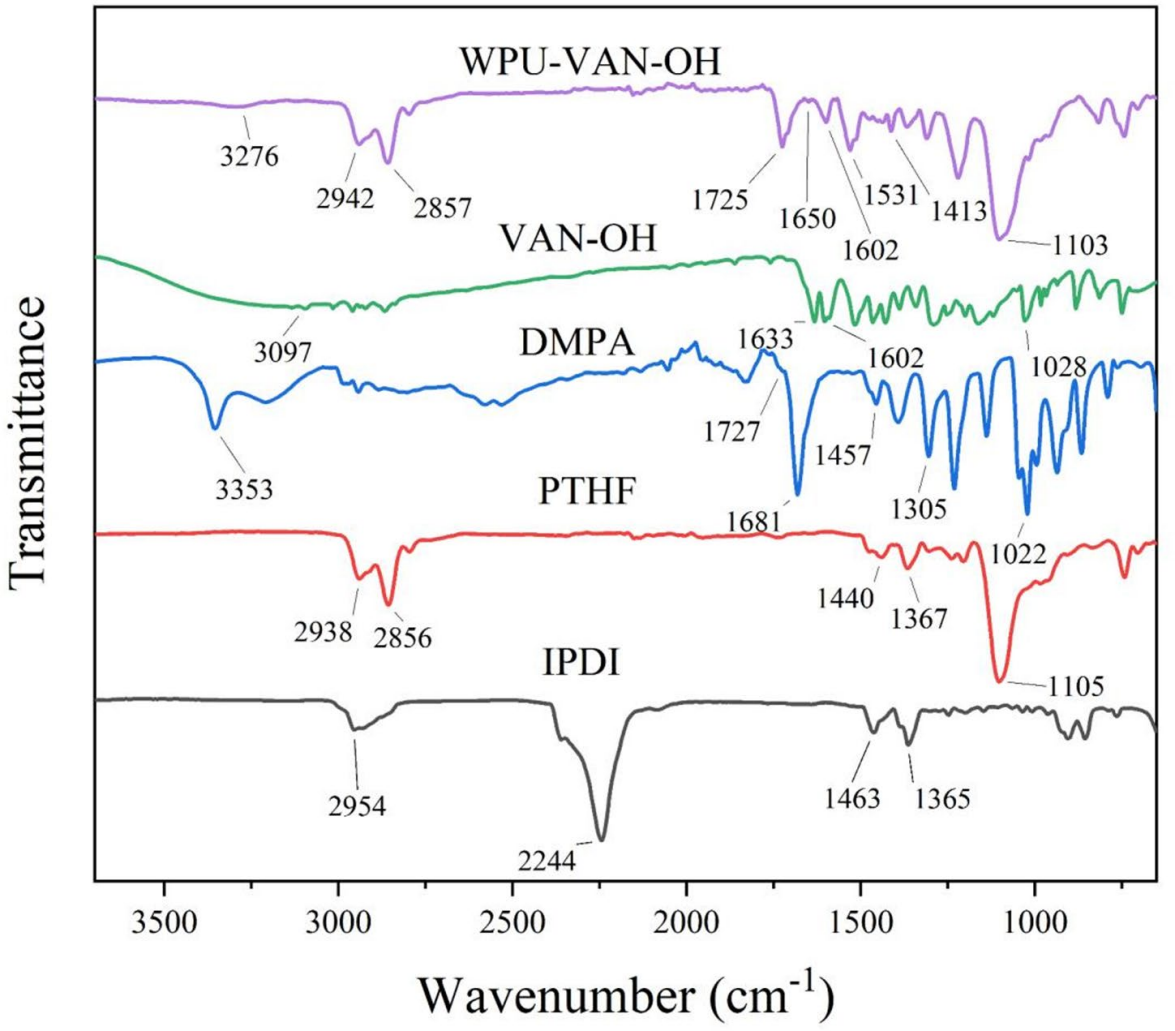
FT-IR analysis of the WPU-VAN-OH film.

The FT-IR spectrum of WPU-VAN-OH exhibits a band at 1103 cm⁻¹, corresponding to C-O-C stretching vibrations from ether linkages in the polymer backbone. Bands at 1413 cm⁻¹ (C-H bending in aliphatic groups) and 1531 cm⁻¹ (N-H bending in urethane bonds) further validate the aliphatic and urethane functionalities. A strong carbonyl (C = O) stretching band at 1725 cm⁻¹ confirms urethane bond formation, while bands at 2857 cm⁻¹ and 2942 cm⁻¹ are attributed to C-H stretching in methylene groups. A broad band at 3276 cm⁻¹ reflects overlapping N-H and O-H stretching vibrations, likely arising from residual hydroxyl groups. The absorption band observed at ~ 1602 cm⁻¹ is attributed to the aromatic C = C stretching vibration of the vanillyl group. This assignment is supported by the fact that the same band appears in the spectrum of VAN-OH. In contrast, the distinct band at ~ 1650 cm⁻¹, which is absent in VAN-OH alone, falls within the characteristic range of C = N stretching vibrations. This feature provides clear evidence for the presence of imine linkages formed through the Schiff-base reaction in WPU-VAN-OH film. These spectral features collectively confirm the successful integration of ether bonds, urethane linkages, and residual hydroxyl groups into the WPU structure (Fig. 5).

The presence of imine (C = N) functionality in VAN-OH and the absence of unreacted isocyanate bands in WPU validate the structural integrity of the synthesized materials. The FT-IR analysis thus provides robust evidence for the chemical composition and successful synthesis of the polyurethane system.

**Fig. 6:**
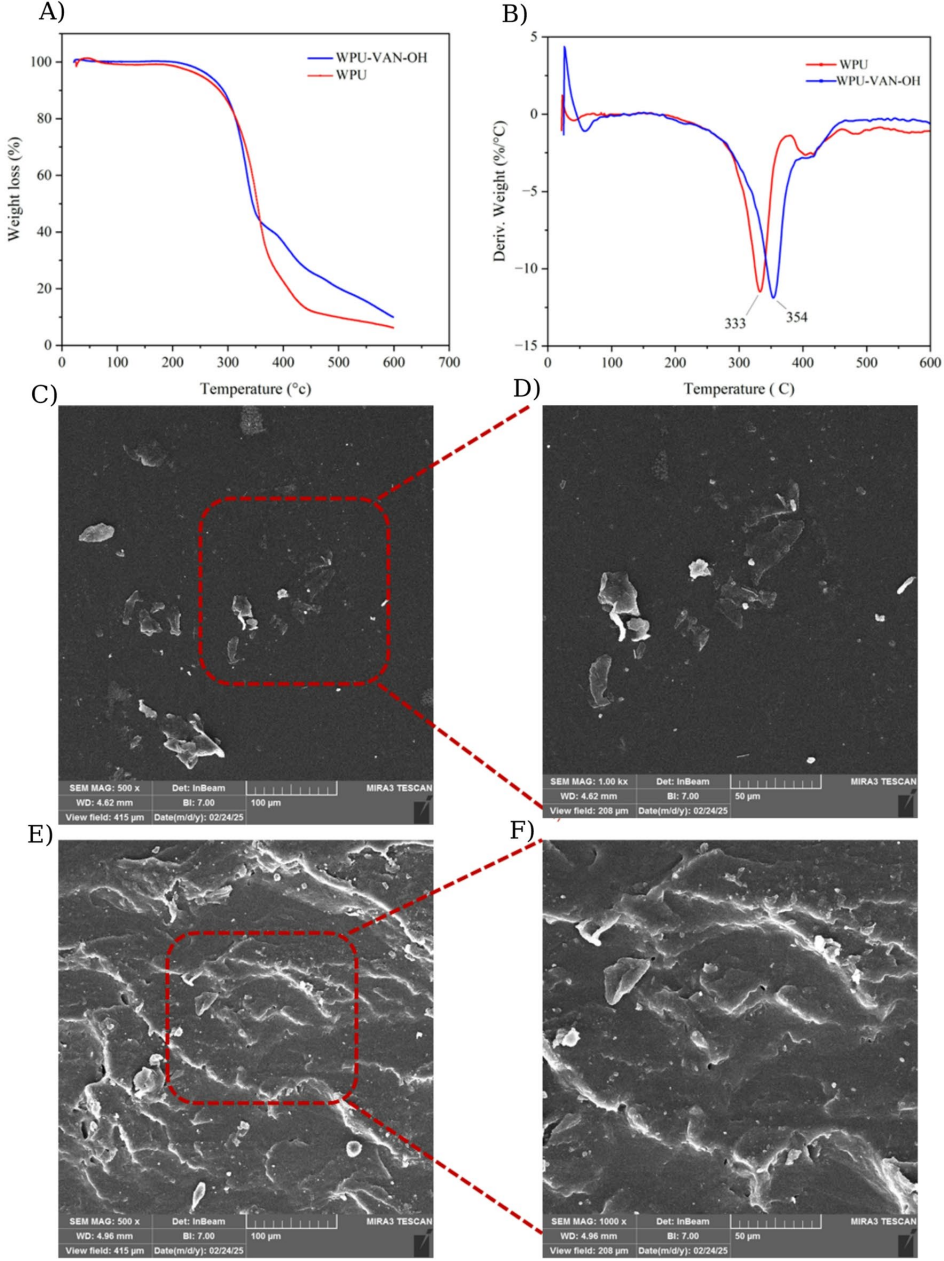
(**A**) TGA curves and (**B**) DTG curves of WPU and WPU-VAN-OH films for thermal degradation analysis. (**C**, **D**) SEM images of WPU film surface morphology. (**E**, **F**) SEM images of WPU-VAN-OH film showing structural features.

Thermal degradation behavior, evaluated by TGA/DTG (Fig. 6A, B), further highlights the advantages of VAN-OH integration^58^. Both films begin degrading above 200 °C, but WPU-VAN-OH displays superior thermal resistance, retaining structural integrity at higher temperatures^59^. This improvement stems from VAN-OH’s role in strengthening intermolecular interactions. The DTG curves reveal an initial degradation at 333 °C for WPU, attributed to the breakdown of urethane linkages and CO₂ release. A subsequent degradation step corresponds to the decomposition of PTHF polyol. In WPU-VAN-OH, this degradation shifts to higher temperatures, which can be ascribed to the presence of aromatic moieties and the intermolecular interactions such as hydrogen bonding and π–π stacking. In contrast, the ester groups, known for their relatively facile degradability, do not contribute to the improved thermal resistance. The enhanced thermal stability of WPU-VAN-OH highlights its superior resistance to thermal stress, making it more suitable for high-temperature applications.

The surface morphology of WPU (Fig. 6C, D) and WPU-VAN-OH films, analyzed via SEM (Fig. 6E, F), reveals significant differences in structural characteristics. Unlike the smooth and uniform surface of the WPU film, adding VAN-OH creates a noticeably rougher texture. This roughness results from increased hydrogen bonding and the formation of extra-hard segments within the material. The roughened surface provides mechanical interlocking sites that greatly enhance adhesion. Notably, this change happens without causing phase separation, proving VAN-OH’s ability to effectively modify surface properties for better functionality.

**Fig. 7:**
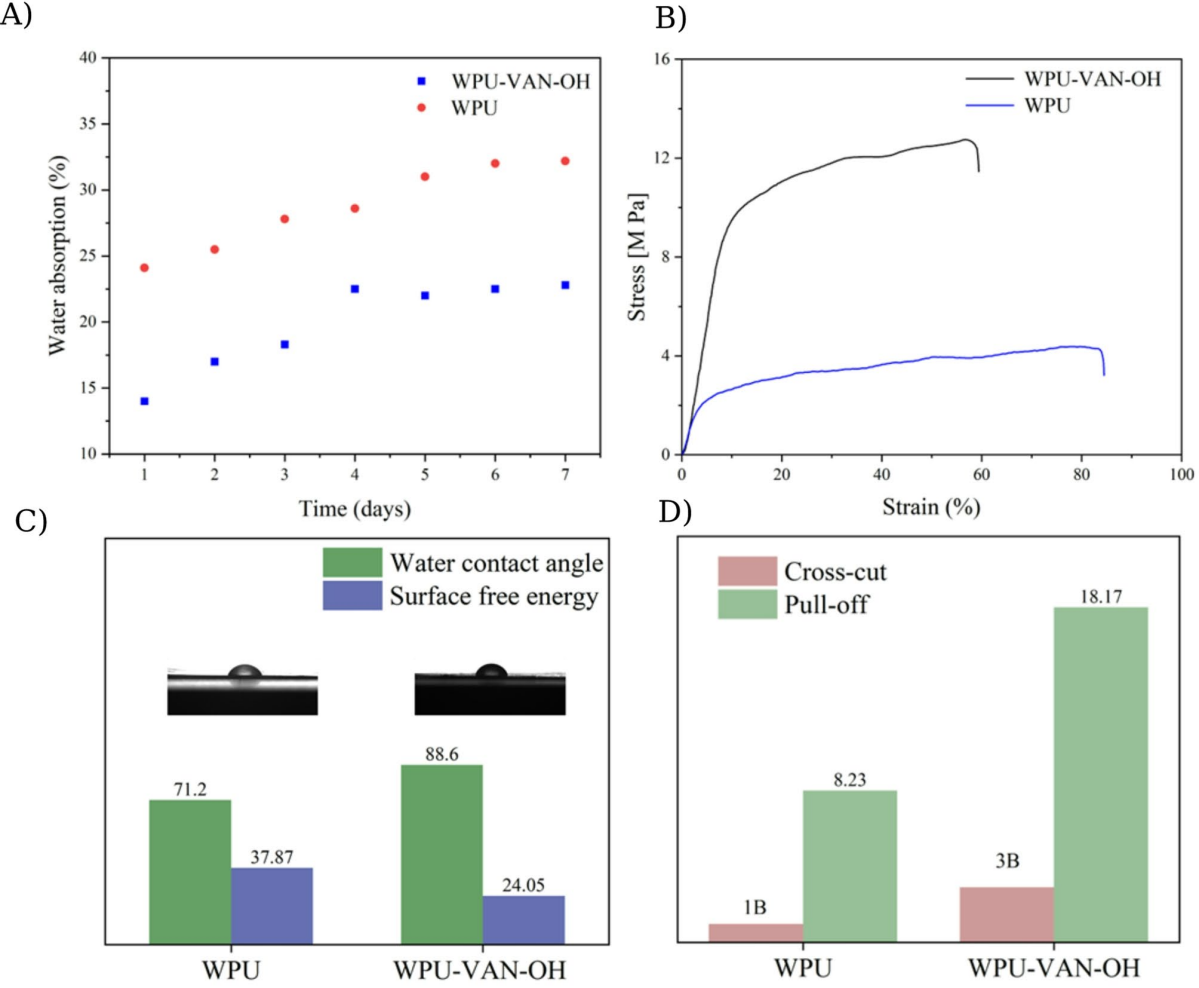
Comparison of WPU and WPU-VAN-OH films showing (**A**) water uptake, (**B**) tensile properties (stress–strain curves), (**C**) water contact angle and surface free energy, and (**D**) adhesion performance (cross-cut and pull-off tests).

The water uptake behavior of WPU and WPU-VAN-OH was investigated over a one-week period. Figure 7A illustrates the swelling trends of both films, highlighting a significant reduction in water absorption in the modified polyurethane^60^. On day 1, WPU exhibited a swelling ratio of approximately 24%, whereas WPU-VAN-OH showed a significantly lower swelling of 14%, marking a 42% reduction in water uptake compared to WPU. By day 7, WPU continued to absorb water, reaching 32.2%, while WPU-VAN-OH stabilized at 22.8%, demonstrating a 30% decrease in long-term swelling. The reduced swelling in WPU-VAN-OH can be attributed to the increased hydrophobicity introduced by the VAN-OH segments in the polyurethane chain. The incorporation of vanillin-based units, which contain aromatic rings and Schiff base (-C = N-) linkages, enhances the overall hydrophobic character of the polymer compared to conventional aliphatic chain extenders commonly used in WPU formulations. Moreover, the presence of vanillin-based segments strengthens the π–π stacking interactions and hydrogen bonding within the hard phase, leading to a more compact and rigid polymer network that restricts water penetration^61^.

The mechanical properties of WPU and WPU-VAN-OH films were assessed via stress-strain analysis to determine the influence of VAN-OH on the tensile characteristics and structural integrity of the polyurethane films^62^. Figure 7B illustrates a significant enhancement in the mechanical strength of WPU-VAN-OH relative to the WPU. WPU-VAN-OH demonstrated a tensile strength of 12.8 MPa, nearly three times higher than WPU’s 4.3 MPa. This substantial improvement suggests that VAN-OH, when used as a chain extender, enhances intermolecular interactions. As a result, it creates a stronger polymer matrix that is better equipped to resist high pressures. The strain at break diminished from 85% in WPU to 60% in WPU-VAN-OH, signifying a decline in flexibility and an augmentation in material stiffness. This effect is due to the existence of aromatic rings and Schiff base connections, which augment π–π stacking interactions and facilitate greater hydrogen bonding inside the polymer network’s hard segments. Mechanical testing revealed a strength–flexibility trade-off upon VAN-OH incorporation. WPU showed ε_b_ ≈ 85.1% and σ_b_ ≈ 4.3 MPa, while WPU-VAN-OH showed ε_b_ ≈ 60.9% and σ_b_ ≈ 12.8 MPa. The substantial increase in tensile strength, with only a moderate reduction in ε_b_ (still > 60%), suggests that the dynamic imine-based network efficiently reinforces the film while maintaining appreciable flexibility, an advantageous balance for outdoor coating applications. Although molecular weight can strongly affect the mechanical performance of polyurethanes, the synthesis conditions (monomer ratios, catalyst, and reaction time) were kept identical for both WPU and WPU-VAN-OH. Therefore, significant variations in molecular weight are not expected. The enhancement in mechanical properties is thus primarily attributed to the incorporation of VAN-OH and the formation of dynamic imine bonds^63,64^. These structural alterations limit chain mobility, resulting in enhanced dimensional stability and decreased elongation under stress. With increased tensile strength and reduced strain, VAN-OH reinforces the polymer matrix, making WPU-VAN-OH more suitable for applications that require high mechanical durability, resistance to deformation, and improved structural integrity.

Water contact angle and surface free energy measurements were employed to evaluate the wettability and surface characteristics of both unmodified WPU and VAN-OH-modified films (Fig. 7C). The modified film showed a marked increase in water contact angle from 71.2° to 88.6°, alongside a reduction in surface free energy from 37.87 mJ/m² to 24.05 mJ/m². These results clearly demonstrate that incorporating VAN-OH enhances the hydrophobicity of the polyurethane film. The rise in water contact angle indicates diminished wettability, which can be ascribed to structural alterations induced by VAN-OH. The incorporation of aromatic rings and Schiff base connections in WPU-VAN-OH enhances the polymer’s overall hydrophobicity. Aromatic systems facilitate π–π stacking interactions, resulting in a denser and less polar surface. This diminishes the capacity of water molecules to form hydrogen bonds with the polymer, thereby elevating the water contact angle. Moreover, Schiff base connections enhance the rigidity of the molecular framework, thereby restricting water-polymer interactions. The reduction in surface free energy from 37.87 mJ/m² in WPU to 24.05 mJ/m² in WPU-VAN-OH further substantiates this discovery. A diminished surface free energy signifies a reduced affinity between the polymer surface and water molecules, hence decreasing interfacial tension and adhesion. This effect is advantageous in applications that require water repellency, decreased fouling, and enhanced resistance to moisture absorption. The reduction in surface free energy indicates that the modified polyurethane possesses a thermodynamically more stable surface, inhibiting excessive water adsorption and degradation over time^65^.

The adhesive capabilities of WPU and WPU-VAN-OH films on stainless steel substrates were tested utilizing cross-cut adhesion (tape adhesion) and pull-off tensile strength tests (Fig. 7D). The findings demonstrate a significant improvement in adhesion for WPU-VAN-OH attributable to its structural alterations and enhanced interfacial interactions with the substrate. The cross-cut adhesion test evaluated adhesion performance on a scale from 0B to 5B, with 0B indicating poor adherence characterized by substantial coating loss, and 5B denoting outstanding adhesion with no detachment. The WPU film demonstrated a 1B rating, with 35–65% of the coating detached, signifying weak to moderate adherence to the stainless-steel substrate. Conversely, the WPU-VAN-OH film attained a 3B rating, with merely 5–15% of the coating detached, indicating a notable enhancement in adhesive strength. The improved adhesion in WPU-VAN-OH is due to the presence of aromatic rings and Schiff base linkages, which facilitate π–π stacking interactions and reinforce hydrogen bonding in the polymer’s hard segments. These structural alterations promote a more compact and stable polymer network, enhancing interfacial contact with the substrate. The addition of -OH groups in VAN-OH enhances polar interactions and strengthens adhesion forces at the polymer-substrate interface, thereby diminishing the risk of delamination.

The pull-off tensile strength test corroborated these findings, indicating that the adhesion strength of WPU and WPU-VAN-OH films varied from 8.23 kgf/cm² to 18.17 kgf/cm², respectively. The substantial enhancement in adhesion strength in WPU-VAN-OH indicates the development of a more interlocking and stable interface, hence diminishing interfacial failure under mechanical stress. The results collectively demonstrate that the modification of WPU with VAN-OH markedly enhances adhesion durability and mechanical interlocking, rendering WPU-VAN-OH an optimal selection for coatings necessitating superior adhesion to metallic surfaces, increased mechanical resilience, and enhanced environmental stability^66,67^.

**Fig. 8:**
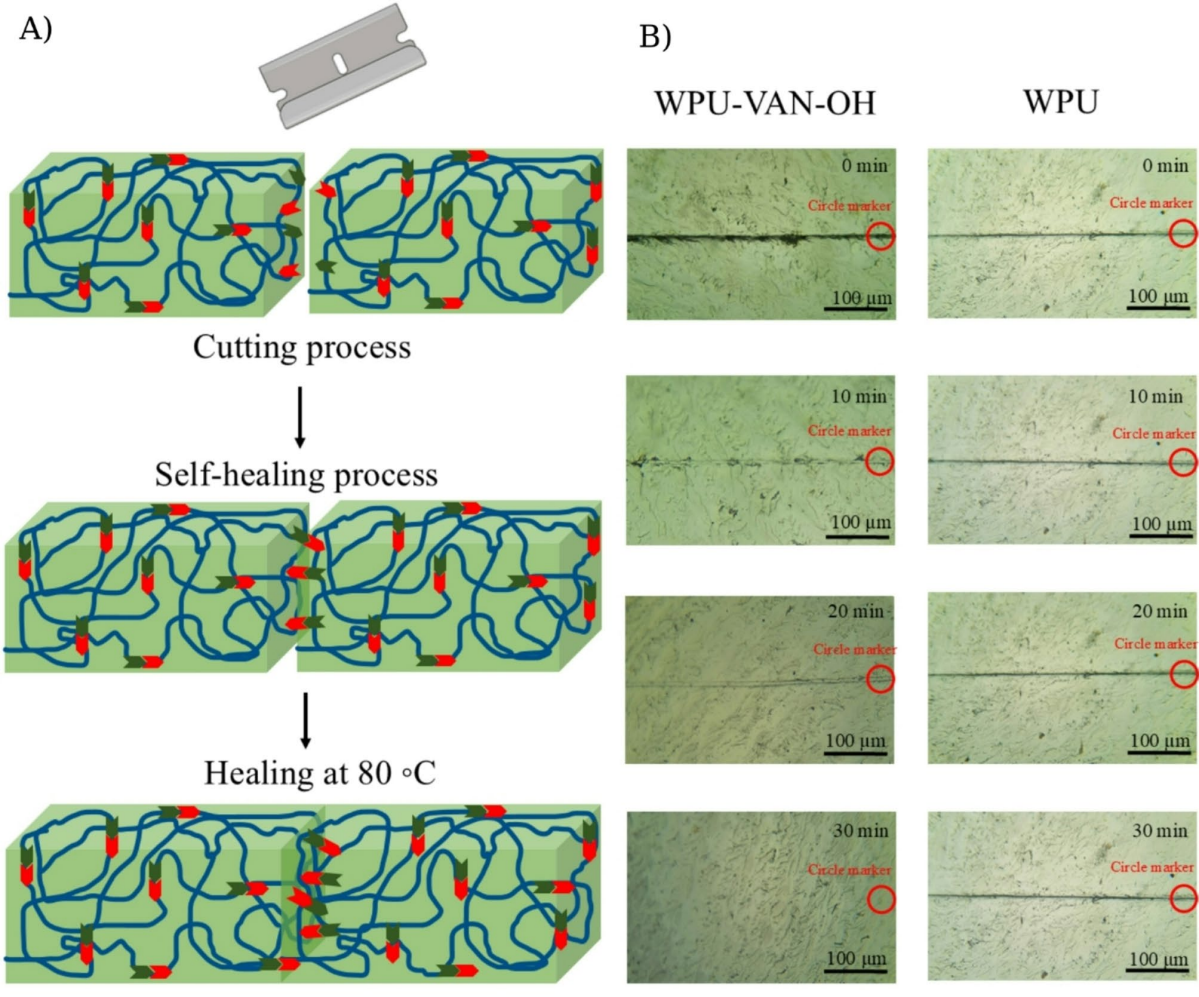
(**A**) Schematic representation of the self-healing mechanism governed by the imine metathesis reaction. (**B**) Optical microscopy images of a scratched pristine sample before and after healing at 80 °C at various time intervals. Red circles indicate the same scratch location throughout the healing process.

Conventional coating materials frequently degrade their integrity when scraped, resulting in diminished functionality and aesthetics. To address this constraint, dynamic covalent chemistry, especially imine bonds, has been extensively utilized because of its reversible characteristics, allowing materials to self-heal structural damage (Fig. 8A). This work evaluated the self-healing properties of WPU-VAN-OH films, which incorporate Schiff base connections, in comparison to traditional WPU films that do not possess these dynamic characteristics^68,69^. The film underwent thermal treatment at a temperature of 80 °C^70–74^. The healing process was observed at designated intervals with a Zeiss Primostar optical microscope fitted with an AmScope 423x ocular camera, yielding comprehensive images of crack closure over time (Fig. 8B). During optical microscopy observations, the samples were fixed on the microscope stage to ensure identical positioning throughout the experiment. Red circle markers were added to the images of both WPU and WPU-VAN-OH to clearly identify the same scratch location at different time intervals. Observations indicated that the WPU-VAN-OH film demonstrated considerable healing properties. After 10 min, the original 10 μm-wide scrape began to noticeably decrease, although the fissure remained evident. After 20 min, significant recovery was apparent, accompanied by additional diminishing of the scratch mark. Full restoration was accomplished within 30 min, as the affected area was practically imperceptible, signifying effective surface rebuilding. The enhanced healing capacity of the WPU-VAN-OH film is ascribed to the mobility of polymer chains above the glass transition temperature (Tg) and the dynamic exchange reactions of imine bonds. Heating enhances molecular mobility, promoting imine metathesis, which allows for the restoration of disrupted bonds and the gradual closure of the affected region. Initially, chain diffusion begins partial healing; as heating advances, enhanced molecular dynamics expedite the process, resulting in complete crack repair. In contrast, the WPU film, devoid of dynamic imine structures, exhibited no discernible healing under the same conditions. After 30 min at 80 °C, the scratch remained unaltered, indicating that standard polyurethane matrices lacking reversible bonding are incapable of autonomous healing^75^. To quantitatively evaluate the self-healing behavior, tensile tests were conducted on WPU-VAN-OH films before and after the thermal healing treatment. The pristine films exhibited a tensile strength of 12.8 MPa, whereas the healed samples recovered to 9.97 MPa. This corresponds to a self-healing efficiency of ~ 78.5%, demonstrating that the dynamic imine bonds introduced by VAN-OH effectively contribute to the restoration of mechanical strength after damage (Supporting Information, Figure S4).

These findings highlight the essential function of vanillin-derived chain extenders and dynamic Schiff base connections in imparting intrinsic self-healing characteristics to polyurethane coatings. WPU-VAN-OH’s capacity to restore surface integrity via mild heat activation renders it a viable material for applications requiring improved durability, lifespan, and diminished maintenance in adverse environments^76^.

The self-healing behavior of WPU-VAN-OH originates from the presence of dynamic imine bonds, whose density depends on the dosage of VAN-OH. A higher concentration of VAN-OH would, in principle, introduce more reversible linkages and facilitate faster bond exchange, but it could also disturb the balance between hard and soft segments, thereby reducing overall film integrity. In the present work, the VAN-OH content was fixed at the optimized level determined by Taguchi DoE, which provided the best compromise between mechanical performance, adhesion, and self-healing efficiency.

## Conclusion

This research effectively synthesized a bio-based waterborne polyurethane, WPU-VAN-OH, by integrating a vanillin-derived chain extender featuring dynamic imine linkages. The resultant material demonstrated a tensile strength of 12.8 MPa, which is threefold greater than that of traditional waterborne polyurethane (4.3 MPa), and exhibited improved thermal stability, maintaining structural integrity at high temperatures. A distinguishing characteristic of WPU-VAN-OH is its remarkable self-healing ability, which completely restores surface scratches within 30 min at 80 °C via the dynamic interchange of imine bonds. These exceptional qualities were attained by meticulous optimization of the DoE methodology, underscoring the essential importance of precise synthesis control in customizing material performance. WPU-VAN-OH, based on renewable vanillin and dynamic imine chemistry, offers enhanced strength, self-healing, and sustainability. It is worth noting that the dosage of VAN-OH is expected to modulate the density of dynamic imine bonds and, consequently, the self-healing efficiency. While this study focused on the optimized composition, a systematic variation of VAN-OH content would provide deeper insights into the structure–property relationship and represents an interesting direction for future research. This study highlights its potential as a high-performance, eco-friendly material for advanced industrial applications.

## Supplementary Information

Below is the link to the electronic supplementary material.

Supplementary Material 1

## Data Availability

Data is provided within the manuscript and supplementary information files.
