# Supplementary material for: Biobased self healing waterborne polyurethane with vanillin derived dynamic Imine bonds for enhanced mechanical strength and performance
Source: Sci Rep. 2025 Sep 26;15:33255. doi: 10.1038/s41598-025-18911-0 (PMC12475146; doi:10.1038/s41598-025-18911-0)
Supplement: Supplementary file 1 — Supplementary Material 1 [file 41598_2025_18911_MOESM1_ESM.docx]

Supporting Information

Biobased Self Healing Waterborne Polyurethane with Vanillin Derived Dynamic Imine Bonds for Enhanced Mechanical Strength and Performance

Ali Reza Banan,* Seyed Mojtaba Keshavarz

Department of Organic Chemistry, Imam Hossein University, Tehran, Iran

*Corresponding author: E-mail: bananjahromi@yahoo.com


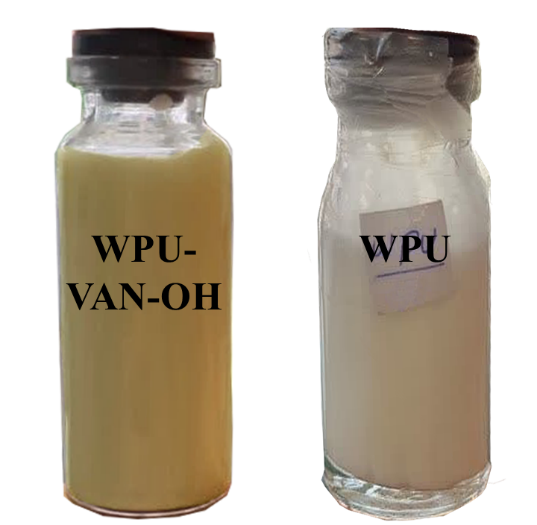


**Figure S1.** Visual comparison of WPU (milky) and WPU-VAN-OH (light-yellow).


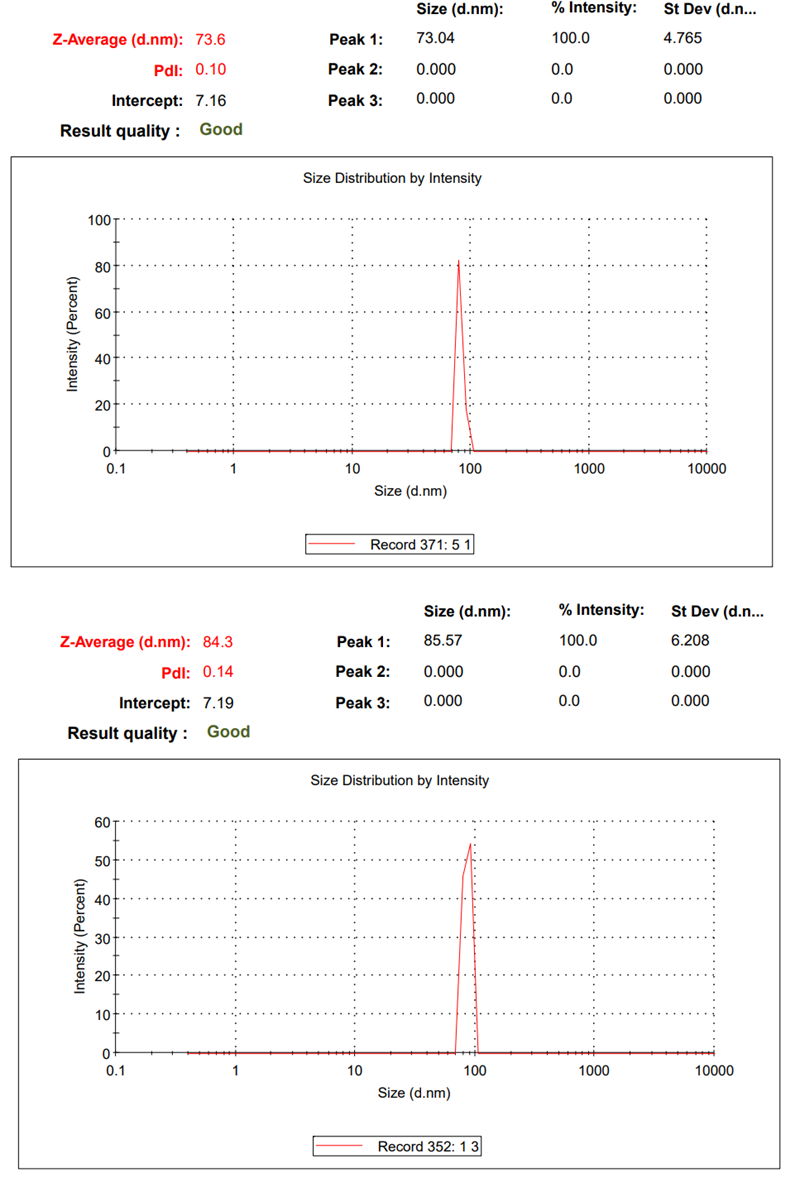


**B)**

**A)**

Figure S2. A) Particle size and PDI of the WPU measured during the first week after synthesi. B) Particle size and PDI of the WPU analyzed in the third month after synthesis.


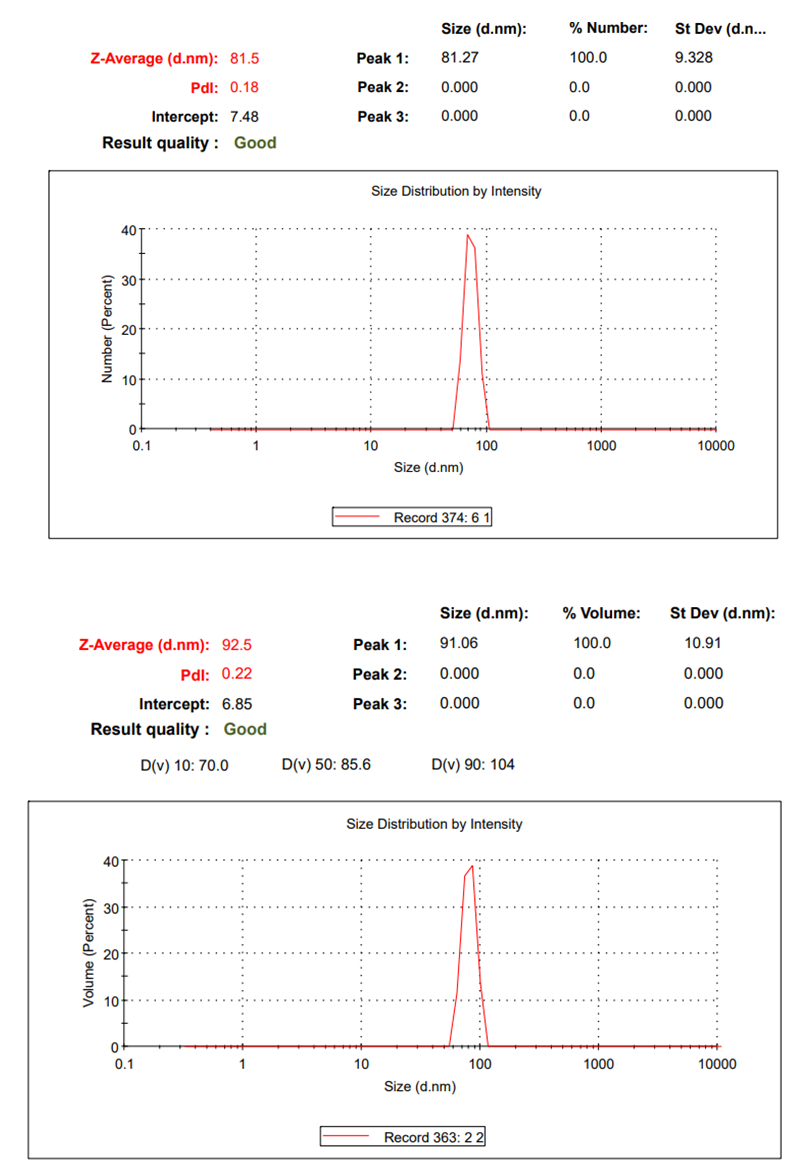


**B)**

**A)**

Figure S3. A) Evaluation of the particle size and PDI of WPU-VAN-OH in the initial week following synthesis. B) Long-term assessment of the particle size and PDI of WPU-VAN-OH conducted three months post-synthesis.

To quantitatively evaluate the self-healing efficiency, tensile tests were performed before and after thermal healing. The initial tensile strength of WPU-VAN-OH films was measured at 12.7 MPa. After subjecting the films to the healing protocol at 80°C, the tensile strength recovered to 9.9 MPa, corresponding to a healing efficiency of ~78.5%. This value is in good agreement with other reported imine-based self-healing polyurethane systems, where healing efficiencies of 70–85% have been typically observed [Refs]. These results further confirm that the incorporation of VAN-OH provides an effective dynamic network capable of mechanical recovery upon heating.


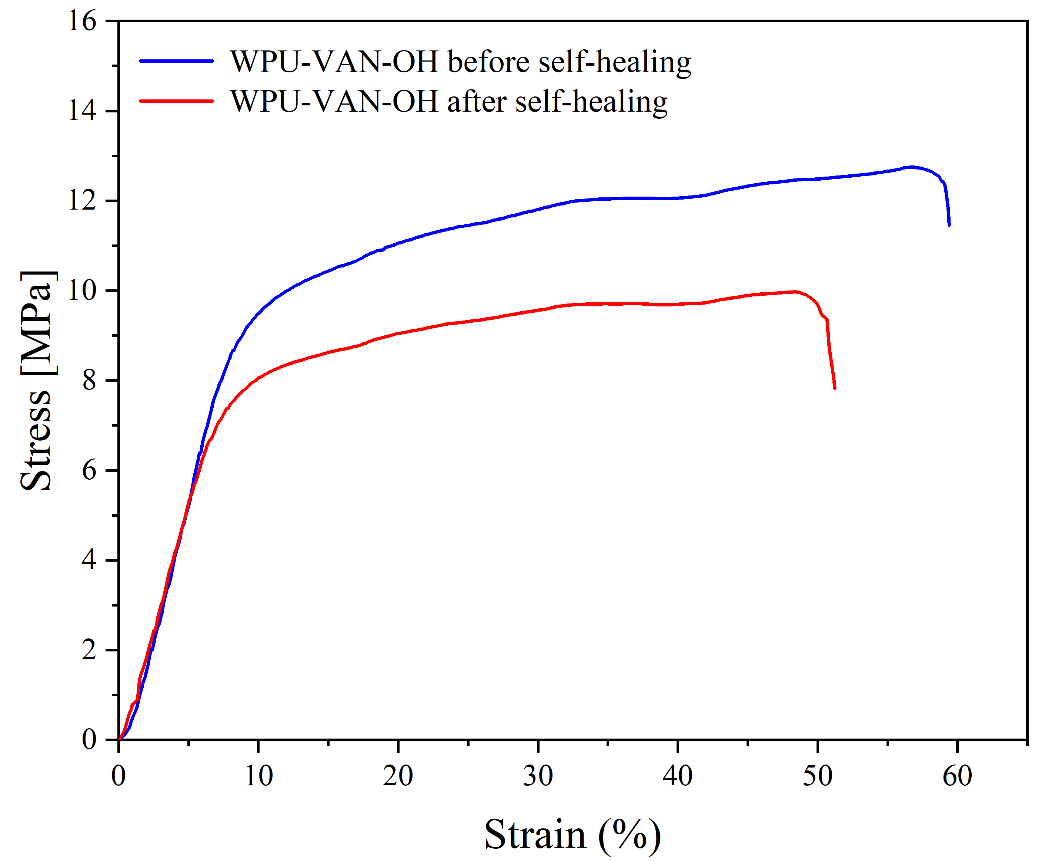


Figure S4. Tensile strength of WPU-VAN-OH films before and after thermal healing at 80 °C.
